# A Calibration Procedure for Field and UAV-Based Uncooled Thermal Infrared Instruments

**DOI:** 10.3390/s20113316

**Published:** 2020-06-10

**Authors:** Bruno Aragon, Kasper Johansen, Stephen Parkes, Yoann Malbeteau, Samir Al-Mashharawi, Talal Al-Amoudi, Cristhian F. Andrade, Darren Turner, Arko Lucieer, Matthew F. McCabe

**Affiliations:** 1 Water Desalination and Reuse Center, King Abdullah University of Science of Technology, Thuwal 23955, Saudi Arabia; Kasper.Johansen@kaust.edu.sa (K.J.); stephen.parkes@intrax.com.au (S.P.); yoann.malbeteau@kaust.edu.sa (Y.M.); Samir.Mashharawi@kaust.edu.sa (S.A.-M.); talal.amoudi@kaust.edu.sa (T.A.-A.); cristhian.andradecorona@kaust.edu.sa (C.F.A.); matthew.mccabe@kaust.edu.sa (M.F.M.); 2 Discipline of Geography and Spatial Sciences, College of Sciences and Engineering, University of Tasmania, Hobart, TAS 7001, Australia; darren.turner@utas.edu.au (D.T.); Arko.Lucieer@utas.edu.au (A.L.)

**Keywords:** thermal infrared camera, calibration, vignetting, UAV, agricultural monitoring, Apogee SI-111, FLIR A655sc, TeAx 640, Tau 2, RPAS

## Abstract

Thermal infrared cameras provide unique information on surface temperature that can benefit a range of environmental, industrial and agricultural applications. However, the use of uncooled thermal cameras for field and unmanned aerial vehicle (UAV) based data collection is often hampered by vignette effects, sensor drift, ambient temperature influences and measurement bias. Here, we develop and apply an ambient temperature-dependent radiometric calibration function that is evaluated against three thermal infrared sensors (Apogee SI-11(Apogee Electronics, Santa Monica, CA, USA), FLIR A655sc (FLIR Systems, Wilsonville, OR, USA), TeAx 640 (TeAx Technology, Wilnsdorf, Germany)). Upon calibration, all systems demonstrated significant improvement in measured surface temperatures when compared against a temperature modulated black body target. The laboratory calibration process used a series of calibrated resistance temperature detectors to measure the temperature of a black body at different ambient temperatures to derive calibration equations for the thermal data acquired by the three sensors. As a point-collecting device, the Apogee sensor was corrected for sensor bias and ambient temperature influences. For the 2D thermal cameras, each pixel was calibrated independently, with results showing that measurement bias and vignette effects were greatly reduced for the FLIR A655sc (from a root mean squared error (RMSE) of 6.219 to 0.815 degrees Celsius (℃)) and TeAx 640 (from an RMSE of 3.438 to 1.013 ℃) cameras. This relatively straightforward approach for the radiometric calibration of infrared thermal sensors can enable more accurate surface temperature retrievals to support field and UAV-based data collection efforts.

## 1. Introduction

Thermal infrared thermometers measure the surface brightness temperature of an object without physical contact by absorbing the infrared radiation onto a thermal detector element. The absorbed radiation changes the electrical resistance of the detector and is then transformed into an electrical signal from which temperature can be inferred [1]. The most common thermal detectors are microbolometers due to lower cost, ease of integration into conventional electronic manufacturing processes and because their large temperature coefficients result in a wide range of resistance changes with radiation absorption [2]. The large resistance change implies that microbolometers have the capacity to detect fine scale radiation changes, which translate into small temperature variations, making them an ideal thermometer. With advancements in semiconductor manufacturing technologies over the last decades, it has become possible to assemble a set of individual thermal infrared thermometers into an array located at the focal plane (FPA) of an imaging system [3]. This array of instruments captures the emitted radiation of the surface or object of interest and transforms the received energy into an intensity map to determine a temperature distribution called infrared thermography (IRT). IRTs offer various advantages to activities requiring temperature monitoring such as non-contact and non-destructive two-dimensional sampling, and they can be used for real-time applications [4].

Due to the aforementioned advantages, IRTs have a wide area of application in multiple fields. For instance, Hui and Fuzhen [5] used thermal images as a detection method for fault diagnosis in electrical equipment; Marino et al. [6] used IRT to study heat loss through building envelopes and Jones [7] highlighted the potential use of thermal image analysis as a medical diagnostic tool. Thermal infrared cameras are also installed onboard satellites to measure surface temperature for Earth observation [8]. Land surface temperature (LST) measured from satellite platforms can be used to detect urban heat islands and improve the planning and heat mitigation strategies of megacities [9,10,11]. Additionally, LST is a key variable for monitoring environmental responses to water availability, such as forest fire risk and severity [12,13], evapotranspiration [14,15,16,17] and drought and water stress [18,19,20,21]. However, with pixel sizes in the tens of meters, satellite based LST retrievals do not always reach the fine spatial or temporal resolutions required for many applications, with precision agriculture being a prime example [22].

An alternative to satellite-based LST acquisition is to mount uncooled thermal cameras on unmanned aerial vehicles (UAV) (also referred to as remote piloted aircraft systems, RPAS), which can increase the spatial resolution to the decimeter scale and enable the possibility to perform on-demand flights, even under overcast conditions [23,24]. Indeed, the use of UAV-based thermal imagery offers many advantages for applications that require high spatial resolution and on-demand inspection capabilities. For instance, Quater et al. [25] found that thermal images captured from a UAV are suitable for inspecting photovoltaic plant performance and for detecting panel failure. In recent years, the use of UAV technologies for environmental, agricultural and plant phenotyping applications has been on the rise due to the reduced cost of implementation and the increase in camera resolution and overall spectral quality [26,27]. For instance, Smigaj et al. [28] found that miniature thermal cameras were capable of representing the spatial and temporal variation of canopy temperature in conifers for stress detection. Rud et al. [29] used IRT images to derive a crop water stress index (CWSI) that was capable of identifying within-field variability of water availability because of different irrigation treatments. In another application, Hoffmann et al. [23] used IRT from a camera mounted on a fixed wing UAV to retrieve field evapotranspiration, an indication of crop water use, using the two-source energy balance model. Another novel use of thermal information comes from the field of wildlife monitoring; the thermal signature of animals can be distinguished from vegetation, which enables applications for animal tracking, counting or conservation purposes [30]. Lhoest et al. [31] developed an automated algorithm that uses thermal images acquired from an UAV platform to count hippopotamuses achieving an accuracy that is comparable with manual delineations.

For UAV applications, uncooled thermal cameras are required due to their smaller size and lower weight. However, the use of uncooled thermal cameras have inherent challenges that must be overcome before they can be used for monitoring purposes, given that the accuracy of the images is regularly required to be within 1 degrees Celsius (°C) [32]. Some of these challenges are spatial non-uniformity of the acquired temperatures on the images, sensor drift (i.e., measured temperature changes while the object’s temperature remains constant due to uncompensated FPA temperature), stabilization of the FPA temperature, and measurement bias that is presented as an offset from the actual target temperature [24,32,33,34,35,36,37,38]. All of the aforementioned challenges make the uncorrected IRT measurements unsuited for applications requiring accurate and stable measurement. For these reasons, camera calibration is necessary to achieve good results during UAV thermal surveys.

One approach to camera calibration is to use ground reference sources to compensate for measurement bias. Torres-Rua [33] proposed a vicarious calibration approach using a black body reference placed within the field of view of the UAV flight trajectory to post-process the thermal images to increase the absolute accuracy of the IRT. Similarly, Pestana et al. [39] used melting snow as a constant 0 °C ground reference to correct the bias in the UAV thermal imagery. However, these approaches do not account for non-homogeneity effects that introduce unrealistic temperature distributions in the form of a vignette effect. Kelly et al. [24] highlighted that the effect of the non-uniformity corrections (NUC) performed by the camera manufacturers during acquisition time was not evident on vignetting reduction. IRT inaccuracies have also been linked to the FPA temperature [34,37,40]. Dhar et al. [41] aimed to correct for the FPA temperature dependency with their calibration approach using a polynomial fit approach in which a cubic drift-correction term was deemed the best for their camera. Ribeiro-Gomes et al. [34] analyzed a linear, polynomial and a neural network calibration approach in which the neural network performed the best, reducing the overall measurement error to <1.5 °C. Additionally, it was found that the calibration approach of Ribeiro-Gomes et al. [34] enabled an easier vicarious calibration post-processing step. However, the actual FPA temperature is often not available to the end user, which makes it impossible to apply the calibration methods that rely on the FPA temperature. Furthermore, uncooled thermal infrared cameras also have an initial stabilization period in which the acquired temperature and corresponding digital numbers deviate from the actual value until the camera has had enough time to stabilize [24,36]. Additionally, thermal cameras can have dead pixels that need to be accounted for, i.e., not all pixels of the camera are functional. Budzier and Gerlach [42] proposed an iterative approach for dead pixel identification, which is carried out after the calibration process. In a recent study, Gonzalez-Chavez et al. [43] proposed an alternative method based on a Planck curve and polynomial regression. The approach was directed towards UAV surveys and required six input parameters, including ambient temperature, humidity levels and surface emissivity. Expanding their previous research, Papini et al. [44] developed a radiometric calibration that estimates gain and offset parameters from thermal images. Using an iterative approach and a set of successive images taken at two blur levels yielded images with root mean squared error (RMSE) of less than 1.6 °C.

It is important to note that larger cameras have also been used for field IRT measurements, both as handheld cameras for field measurements and mounted in autonomous vehicles. Deery et al. [45] used a FLIR SC645 (FLIR Systems, Wilsonville, OR, USA) handheld camera (which has equivalent technical characteristics to the one used in this study) adapted for use with a small helicopter, e.g., for phenotyping applications in which lower canopy temperatures might be associated with better genetic gains and a sign of higher stomatal conductance. Zhang et al. [46] developed a 3D robotic system for high throughput phenotyping using the FLIR A655sc camera, enabling on-demand diurnal analysis over a controlled agricultural environment for precision agricultural studies. Additionally, as a low-cost alternative, cheaper handheld radiometers are also used for point-based measurements. Mahan and Yeater [47] highlighted that the Apogee radiometers provide accurate field based IRT measurements. Indeed, the Apogee radiometers are routinely used as a validation source for satellite and UAV-based studies that involve retrieval of LST [22,48,49].

The contributions of the present study are twofold. First, we propose an ambient temperature-dependent calibration function that can be applied to a wide variety of infrared radiometers, evaluated here on the Apogee SI-111 infrared radiometer and the FLIR A655sc and TeAx 640 (based on a Tau 2 core) uncooled thermal cameras. To achieve this goal, we developed an accurate temperature reading based on resistance temperature detectors (RTDs) attached to a black body and suitable for a temperature range between 0–60 °C. This reference black body was placed in an environmental chamber to emulate different field conditions. Second, we apply the developed calibration function to correct for the vignette effect on the acquired IRT images. This objective was a by-product of the calibration function by applying it on a pixel-by-pixel basis. The vignetting reduction was later verified by analyzing IRT images for different target black body temperatures and at different ambient temperatures. Our research provides a novel approach to radiometric calibration of IRT data by incorporating measurements of ambient temperature, as a proxy of FPA temperature, which is generally not available. The proposed calibration approach has implications for future projects that require the acquisition of highly accurate thermal information across various disciplines and will benefit applications in natural ecosystem monitoring, precision agriculture, hydrology, urban planning among other fields. Finally, we provide a suggested workflow to ensure accurate thermal infrared field data collection and discuss other considerations for data post-processing.

## 2. Materials and Methods

Here, we propose a novel approach to develop an ambient temperature-dependent calibration function for infrared radiometers. Special care was taken to acquire a precise reference temperature measurement during the laboratory calibration process, which was carried under four different ambient temperature conditions that emulate common field conditions and later evaluated against three thermal infrared sensors (Apogee SI-11, FLIR A655sc, TeAx 640).

### 2.1. Thermal Instruments

**Figure 1:**
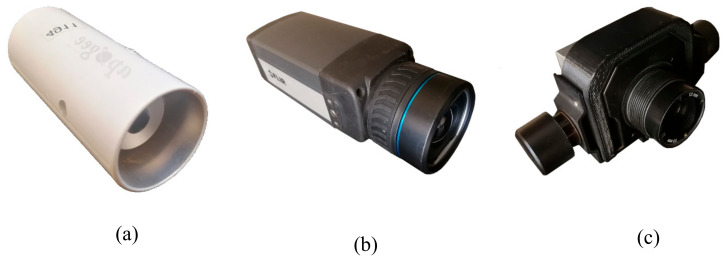
Instruments calibrated during this study (**a**) Apogee SI-111 infrared radiometer, (**b**) FLIR A655sc uncooled radiometric thermal camera, (**c**) TeAx 640 miniature uncooled radiometric thermal camera for unmanned aerial vehicle (UAV) applications.

This study analyzed three different thermal infrared sensors. The first instrument was the Apogee SI-111 infrared radiometer (Apogee Instruments Inc., Logan, UT, USA, Figure 1a). The spectral range of this sensor is from 8 to 14 μm, with an operating temperature range from −40 °C to 80 °C, a manufacturer accuracy of ±0.5 °C and a measurement range of –60 °C to 110 °C. Apogee radiometers convert a voltage to a temperature reading that is representative of the averaged sensor footprint values [50]. Readings were taken every second.

Next, we analyzed and calibrated the FLIR A655sc uncooled radiometric camera (FLIR System Inc., Wilsonville, OR, USA, Figure 1b) with a resolution of 640 × 480 pixels and a weight of 0.90 kg. The sensor inside the camera has a spectral range of 7.5–14.0 µm, a dynamic range of 16 bits, a scene temperature range of −40 °C to 150 °C (used in this study) and another range of 100 °C to 650 °C and a manufacturer accuracy of ±2 °C or ±2% of the reading [51]. Images were captured every second.

Finally, we also calibrated the TeAx 640 radiometric thermal camera (TeAx, Wilnsdorf, Germany, Figure 1c) designed to be used on UAV platforms. This camera has a resolution of 640 × 512 pixels and a weight of 95 g. The sensor is based on a Tau 2 core [52,53] with a spectral range of 7.5–13.5 µm, a manufacturer accuracy of ±5 °C or 5% of the reading, and a scene temperature range of −25 °C to +135 °C in the recommended high-gain mode (used in this study) and of −40 °C to +550 °C in the low-gain mode [53]. The TeAx 640 camera performs non-uniformity corrections every 100 frames, but the effect of these corrections was not evident, as the images still presented significant vignette effects. The capture rate of the TeAx camera is 8.33 Hz (i.e., one image every ~120 ms) and frames were extracted to the closest second (i.e., taking the frame number that corresponds to ~1 s intervals) for consistency between all instruments and black body reading synchronization. For the TeAx 640, the digital numbers were converted to Celsius brightness temperature values with the following conversion formula applicable for the Tau 2 core inside the camera [54].

(1) $$T_{brightness}=DN\times 0.04-273.15$$

### 2.2. Calibration Workflow

**Figure 2:**
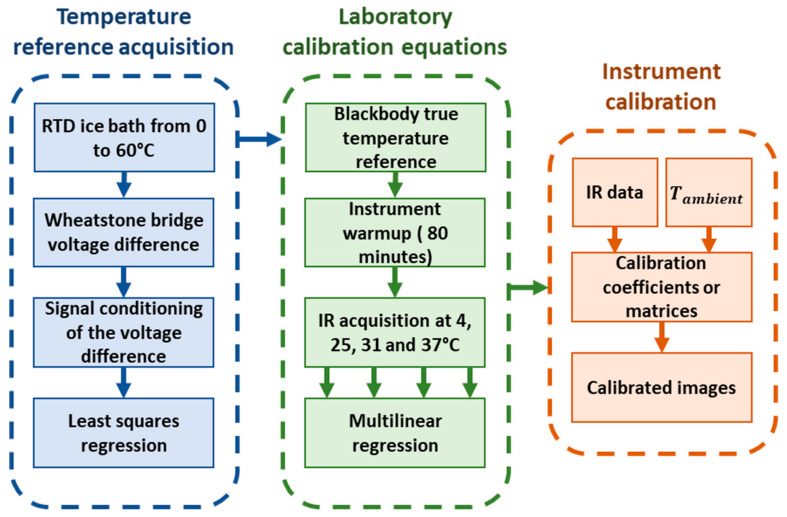
Schematic of the calibration workflow. The first step was to produce a reliable temperature reference and then collect infrared temperature information at multiple temperatures in an environmental chamber to fit the data to the temperature reference using multilinear regression (MLR) to produce calibration equation matrices. Finally, these calibration matrices were applied to the uncalibrated thermal infrared data. This approach corrects for the ambient temperature effect as well as non-uniformity for each individual pixel in the image.

A three-step process was applied to develop a temperature dependent calibration function for infrared radiometers. As a first step, a series of resistance temperature detectors (RTDs) were calibrated across a temperature range from 0 °C up to 60 °C using an ice bath that was gradually heated. These RTDs were attached to the back-plate of the FLIR 4 black body and were connected to a data acquisition card to record the temperature at regular one-second intervals. This setup ensured a consistent and highly precise temperature recording for the calibration of three distinct thermal instruments, i.e., the Apogee SI-111 infrared radiometer, the FLIR A655sc and TeAx 640 uncooled thermal cameras. A schematic of the calibration process is depicted on Figure 2.

### 2.3. Black Body Characterization

**Figure 3:**
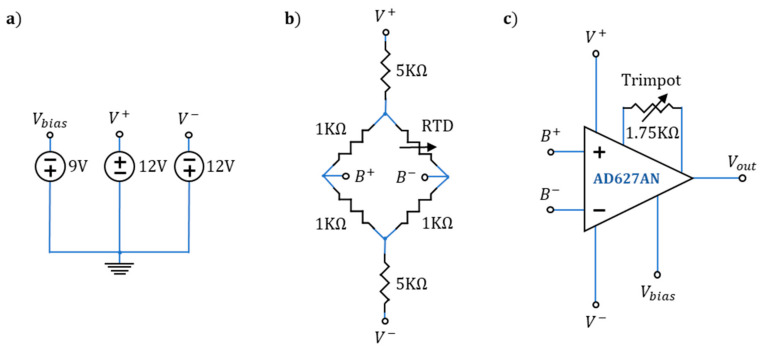
Resistance temperature detector (RTD) sensing circuit (**a**), power source configuration for the Wheatstone bridge and signal conditioning of the RTD signal where $V^{+},\;V^{-}$ and $V_{bias}$ represents a physical point of connection to 12 V, –12 V and 9 V respectively; (**b**), Wheatstone bridge configuration for the two wire RTDs. The circuit is excited by the two voltage sources at $V^{+}$ and $V^{-}$. A change on the RTD nominal value creates a voltage difference between $B^{+}$ and $B^{-}$; (**c**), instrumentation amplifier circuit, the AD627AN amplifies and shifts the value of the differential voltage between $B^{+}$ and $B^{-}$ and outputs a ground referenced voltage $V_{out}$ that is connected to a data acquisition card (Labjack U12) for logging.

To ensure a reliable and known temperature source, we built a set of RTD sensors to measure the black body temperature. Each RTD was based on the PT1000 platinum resistance element that has a nominal electrical resistance value of 1 k Ohms at 0 °C. Each circuit was powered by the voltage source arrangement displayed in Figure 3a, where the labels $V_{bias}$, $V^{+}$, and $V^{-}$ indicate points of physical connection to the other parts of the circuit. The RTDs were connected into an array of 1 k-Ohm resistors (with a 1% error value) called a Wheatstone bridge. The purpose of this electrical circuit was to measure the differential voltage (i.e., the voltage at $B^{+}\;and\;B^{-}$) between the terminals of two resistors powered by the same voltage source in parallel (Figure 3b). The RTD resistance value will be different from the other resistances in the Wheatstone bridge unless the ambient temperature is 0 °C. This difference in resistance due to the ambient temperature gives rise to a voltage differential that is proportional to the RTD value. Additionally, two 5 *k* Ohm resistors were placed in series with the bridge arrangement to avoid RTD self-heating (and a related bias in the voltage measurements) due to the circulating current (Figure 3b). This voltage difference is too small to measure directly, so the Wheatstone bridge arrangement was connected to an instrumentation operational amplifier (AD627AN) for signal conditioning (Figure 3c). The gain and bias of the amplifier were configured to maximize the voltage range of the analog-to-digital converter of the Labjack U12 data acquisition card that takes an analog voltage and transforms it into a digital reading. The voltage range was set to correspond to a temperature range between –10 ℃ and 70 ℃. The Labjack had a ±10 V input range with a 12-bit resolution [55]. Trim potentiometers, a variable resistance that allows for fine adjustments, were used for accurately setting the gain of the amplifier. Before each experiment, the trim potentiometers of the amplifier circuit were offset to 1.75 *k* Ohm. This value was determined to fit the voltage range of the data acquisition card. The bias voltage was also checked before conducting the experiments and set to –9 V, thus ensuring the same gain for every experiment.

For the RTDs to serve as a temperature reference, each of them was individually calibrated inside an ice bath that was heated from 0 °C to 60 °C in a stirring hotplate to ensure even heating. A linear regression was performed on each of the logged RTDs voltage values against observed temperatures from the average of two graduated mercury thermometers that were submerged with the RTDs in the ice bath. Each RTD was found to be linear (as expected), with a coefficient of determination ≥0.99. The calibrated RTDs were mounted on the back-plate of the FLIR 4 black body [56]. The FLIR 4 black body has an emissivity value of 0.95 (FLIR application support, personal communication, 4 February 2020) and a large target area, which allows for a larger measurement distance between the infrared sensors and the surface of the black body. This increased measurement distance reduced the possibility of a temperature bias on the instruments produced by the emitted heat of the black body. The black body heats to a wide range of temperatures in proportion to the applied voltage at its terminals.

### 2.4. Laboratory Setup

Each instrument to calibrate along with the black body and related instrumentation were placed in an environmental chamber (i.e., a properly insulated room equipped with heating and cooling capabilities and control electronics to maintain the ambient temperature at a constant setting). Each experiment was repeated multiple times at varying ambient temperatures to properly account for any possible temperature dependence in the sensors. The different ambient temperatures were 4, 22, 33 and 37 ℃ as the chamber was shared between different research group’s long-term experiments, and so varying the temperatures was not possible. Nevertheless, these temperatures cover a range of commonly encountered field ambient temperatures. The environmental chamber used in this study was designed by Harris Environmental Systems and had floor dimensions of 4 × 3 m. To avoid draft influences, the calibration system was left to record data unattended during the experiment duration. To sense the ambient temperature, an additional RTD was left exposed to the outside temperature and its values were logged along with the black body temperature at the same one-second interval.

Previous studies have found that thermal infrared instruments undergo a warm-up period during which the measurements are erratic [24,57]. To avoid the effects of the warm-up period, we allowed the camera to reach thermal equilibrium with the environmental chamber, and only those measurements recorded after 80 min of operation were considered for the calibration process. The RTDs were configured to sample every second. No smoothing was done to the reference data. It is important to note that the RTD temperature values are actual temperature readings rather than brightness temperatures. Therefore, the emissivity of the black body was used to correct the radiometer measurements prior to the calibration process by dividing the measured value by the emissivity of the black body (0.95).

To reduce bias and self-heating interference due to the emitted heat from the black body, each instrument was placed at a fixed distance such that it could cover the black body’s surface in its field of view. The area of the heating surface of the black body is ~81 cm^2^. The field of view of the Apogee SI-111 was estimated using the equations on the manufacturer’s website [58], and that allowed us to place it at a distance of ~10 cm to the black body. The distance of the FLIR A655sc and the TeAx cameras to the black body was estimated visually from the captured images (i.e., the camera’s distance was evaluated such that the black body’s housing was no longer visible from the real-time camera feed) and determined to be ~8 cm to cover the maximum heating area of the black body.

Each of the experiments lasted for around 50 min, which is the time it took the black body to go from ~60 °C to around ambient temperature. Using the cool down period to collect the temperature measurements (rather than collecting the samples while heating the black body) ensured a gradual temperature reduction that was better suited for our data collection approach without the need for implementing a temperature controller. A complete list of the materials and associated cost to replicate the calibration experiments is presented in Table 1. The most expensive was the environmental chamber, but there are other commercial alternatives and brands that could offer a more cost effective solution.

### 2.5. Derivation of Calibration Equations

We assume that the observed temperatures form a plane in space that could be modelled by means of a multilinear regression (MLR) of the form:

(2) $$T_{cal}=T_{radiometer}^{2}\beta_{3}+T_{radiometer}\beta_{2}+T_{a}\beta_{1}+\beta_{0}$$

where $T_{cal}$ is the calibrated measurement of temperature, $T_{radiometer}$ is the non-calibrated radiometer measurement and $T_{a}$ is the ambient temperature. The parameters $\beta_{3}$, $\beta_{2}$, $\beta_{1}$ and $\beta_{0}$ are determined in such a way that the sum of squares residuals term is at a minimum. With n independent observations, Equation (1) can be organized into a vector and matrix form:

(3) $$T_{cal}=T\beta +\epsilon$$

in which the least square estimate of the linear coefficients β is:

(4) $$\hat{\beta }={\left(T^{\prime }T\right)}^{-1}T^{\prime }T_{cal}$$

and the predicted value $\hat{T_{cal}}$ is then:

(5) $$\hat{T_{cal}}=T\hat{\beta }$$

In this way, the predicted values at different temperatures are given by multiplying the known measured values with the uncalibrated instrument and observed air temperature and the least squares coefficients (i.e., solving Equation (2)). Given the large number of observations of each instrument, a uniform random sampling of the dataset was performed across all the environmental chamber temperatures. This provided 100 samples from each environmental chamber experiment for a total of 400 samples, of which 330 samples were used for training and 70 for evaluation. The sample size was evaluated for the Apogee sensor and a small set of pixels from both thermal cameras by comparing the results from using all available data points and the 400 sample subset, yielding an RMSE and r^2^ within 2% each other. To avoid overfitting of the data and to increase the certainty of the corrected temperature measurements obtained with the regressed parameters, we performed k-fold cross-validation on the sampled dataset. To determine *k*, 5-fold to 10-fold cross-validation procedures were evaluated, for which *k* = 5 was found to be appropriate both in computational cost and convergence of the resulting parameters. The final parameter value is the average of all estimated parameters in the *k* folds to reduce the variance in the final parameter estimate [59]. The choice of the fitting equation was made on three aspects. First, its computational simplicity; second, the ease of interpretation of the resulting calibration coefficients; and third, the ability to study in an indirect way the effects of the ambient temperature on the camera measurements and the vignette effect, which cannot be done with more complex methods (e.g., neural networks, random forest, SVM). Each pixel in the cameras was treated as an independent thermal infrared radiometer.

### 2.6. Calibration Evaluation

The calibrated estimates were evaluated against the black body temperature measured by the RTD installed on the back-plate. The chosen statistics for evaluation are: the coefficient of determination ($r^{2}$), an estimate of the variability explained by the regression; the bias, which describes the over- or underprediction amount of the calibration; and the root mean square error (RMSE).

(6) $$r^{2}={\left(\frac{cov\left(x,y\right)}{\sigma_{y}\sigma_{x}}\right)}^{2}$$

(7) $$bias=\frac{1}{n}\overset{n}{\underset{i=1}{\sum }}\left(y_{i}-x_{i}\right)$$

(8) $$RMSE=\sqrt{\frac{1}{n}\overset{n}{\underset{i=1}{\sum }}{\left(y_{i}-x_{i}\right)}^{2}}$$

where $cov\left(x,y\right)$ is the covariance between the measured values $x_{i}$ and the estimated values $y_{i}$, σ is the standard deviation and n is the number of observations. For the FLIR and TeAx cameras, the vignette effect was evaluated by the interquartile range (IQR), estimated as the difference between the 75th and 25th percentiles and the standard deviation, in which a value close to zero is considered to be better. The IQR and σ of the evaluation sample was done as a mean quantity across all the pixels of the images for the 70 validation samples.

## 3. Results

This section presents the results of the radiometric calibration for the three thermal instruments. First, we present the corrected Apogee measurements. Next, we show the calibration results and vignetting correction of the handheld FLIR 655sc and UAV-based TeAx thermal infrared cameras over the different ambient temperature regimes. To use the proposed MLR methodology on the 2D thermal images of the cameras, we assume that each pixel in the camera is equivalent to an independent sensor and thus will have its own set of calibration coefficients. To correct an image from the thermal cameras, an element wise multiplication of matrices is performed between each term of the MLR equation.

### 3.1. Apogee Calibration

**Figure 4:**
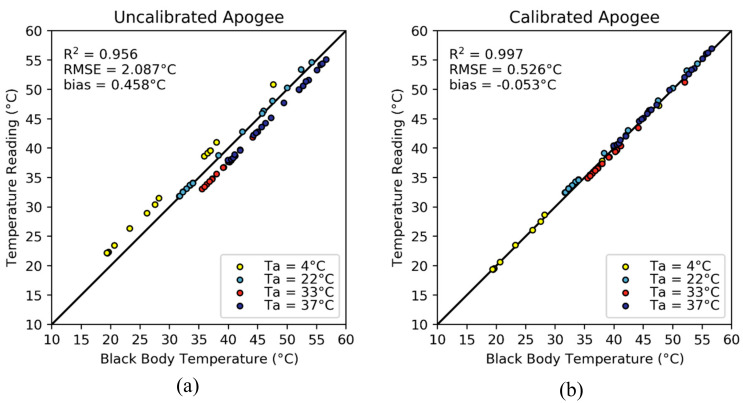
(**a**) A scatterplot of the uncalibrated Apogee measurements taken from inside the environmental chamber at different temperatures. The measurements from the Apogee sensor have a linear relationship with the reference black body temperature, but have a temperature dependent bias. (**b**) The scatterplot after the calibration process, showing reduced measurement bias and an improved RMSE. The plots and the statistics were made from the evaluation dataset (n = 70).

The Apogee radiometer that was calibrated during the laboratory experiments did not meet the manufacturer’s accuracy specifications, showing an overall bias of 0.458 °C and RMSE of 2.087 °C. Even though Apogee radiometers do take into account the sensor temperature when computing the target temperature [60], it was found that the actual temperature varied as a function of the ambient temperature (Figure 3a). However, the instrument measurements remained highly linearly related to black body temperature readings, with an $r^{2}$ of 0.956. This indicates that the Apogee instruments become biased after their initial calibration process. As can be seen in Figure 4a, the uncalibrated temperatures of the Apogee radiometer tracked the actual temperature change of the black body, needing mostly an offset compensation that depended on the environmental chamber temperature. This bias in the temperature measurements by the non-calibrated Apogee radiometer occurred for all ambient temperatures, but was more noticeable at high and low air temperatures where the bias was −2.773, 2.410 and 2.105 °C for air temperature of 4, 33 and 37 °C, respectively. The bias at an ambient temperature of 22 °C was only –0.429 °C.

In Figure 4b, the scatterplot between the black body and the calibrated Apogee temperature measurements shows that the bias of the Apogee radiometer was reduced from 0.458 °C to –0.053 °C, while still maintaining the high linearity. A reduction in the RMSE of 1.561 °C (from 2.087 to 0.526 ℃) was observed and the r^2^ increased from 0.956 to 0.997, which was a significant improvement from the uncalibrated readings. The resulting calibration equation for the Apogee instrument was:

(9) $$T_{cal}=-0.001\cdot T_{radiometer}^{2}+1.020\cdot T_{radiometer}+0.168\cdot T_{a}-3.499$$

As expected, the $\beta_{0}$ calibration coefficient was the largest, indicating that the largest contribution in the calibration process came from the sensor bias. The ambient temperature also influenced the measurements by a 0.168 °C additional offset for every degree increase in temperature. The $\beta_{2}$ calibration coefficient was almost unity, whereas the $\beta_{3}$ coefficient was almost zero, and started to affect the estimates only at high temperatures. This indicates that a linear form of the regression equation could also be a suitable alternative. It is important to note that this equation is unique to a particular sensor, so data collection and calibration experiment must be performed on individual radiometers.

### 3.2. FLIR A655sc Calibration

**Figure 5:**
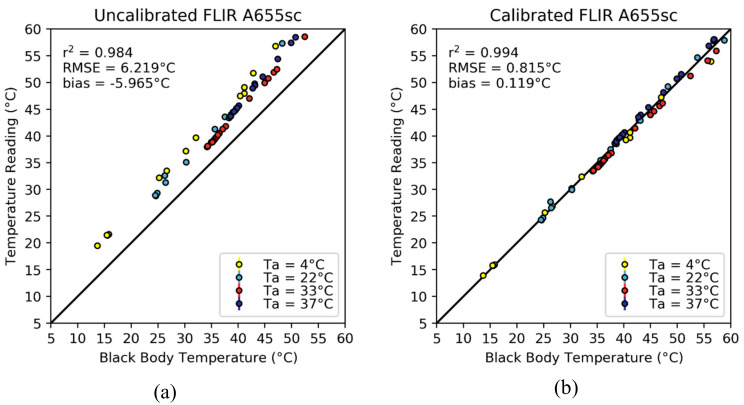
Scatterplot of the average temperature readings of the FLIR A655sc camera before (**a**) and after (**b**) calibration. Error bars are included to show the spread from the mean temperature value of each image and are three times the standard deviation of each observation. However, for the FLIR A655sc, the standard deviation was small and the error bars are visible only for a couple of samples when the black body temperature was ~45 °C. The plots and the statistics were made from the evaluation dataset (n = 70).

To calibrate the FLIR A655sc thermal infrared camera, we applied the same methodology that was described in Section 2.4, but under the assumption that each of the camera pixels was an independent spot radiometer i.e. that the value of each pixel is independent of any of the other pixel values in the thermography. This assumption comes from the FPA structure, in which each microbolometer is replicated and read independently from each other. Before the calibration process, the temperatures measured with the FLIR A655sc camera had a mean bias across all environmental chamber temperatures (4, 22, 33 and 37 °C) of –5.965 °C, an RMSE of 6.219 °C and an r^2^ of 0.984 (Figure 5a). This implied that the camera performed poorly to detect the absolute temperature values and that it was overestimating the target temperature by a large margin (~6 °C).

After the calibration process (Figure 5b), the measured temperature values displayed a better fit with the actual temperature of the black body, although at black body temperatures ≥50 C, there was a larger deviation from the 1:1 line (RMSE of 1.290 °C compared to 0.688 °C when the black body temperature is below 50 ℃). The calibrated measurements of the FLIR A655sc had an overall bias of 0.119 °C, an RMSE of 0.815 C and an r^2^ of 0.994, increasing the r^2^ by 0.01, and decreasing the bias and RMSE by 6.084 ℃ and 5.404 ℃, respectively. To visualize the fitting equation, an average of all the pixel parameters is shown below:

(10) $$T_{cal}=-0.001\cdot T_{radiometer}^{2}+0.998\cdot T_{radiometer}+0.089\cdot T_{a}-5.3134$$

The $\beta_{0}$ coefficient was one of the most significant, showing that on average across all pixels, the camera needed to be corrected for about 5.3 °C. Even though the $\beta_{1}$ coefficient was small (0.089), its influence increased with ambient temperature becoming significant (i.e., contributing 1 °C to the measurement) for each increase of 11.2 °C.

**Figure 6:**
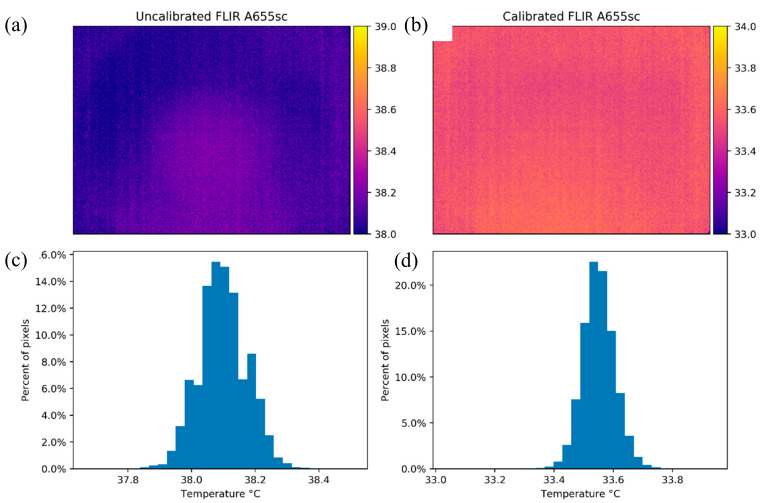
Temperature maps and histograms for the FLIR A655sc camera while looking at the black body with a temperature of 34.28 °C. Panels (**a**) and (**c**) show the results before the calibration process and panels (**b**) and (**d**) depict the results of the same image after applying the calibration equation to each pixel in the whole image.

The vignette effect could not be appreciated on either panel of Figure 5, for which the average σ was 0.109 °C and the IQR was 0.141 °C before calibrating the camera. However, the overall standard deviation and interquartile range decreased to 0.059 °C and 0.078 °C, respectively, after the calibration process. These results mean that the original images did not have a significant vignette effect, but the calibration did improve the images beyond correcting for the bias in the measurements. This improvement is displayed further in Figure 6a, where the temperature map of the uncalibrated image is shown. The pixel values across the image are mostly homogeneous and within less than a degree from the mean value, as is corroborated by the corresponding histogram (Figure 6c). Indeed, σ was 0.076°C and the IQR was 0.041 °C for this specific image, indicating small deviations from the average temperature value. Nevertheless, the average bias from the true black body temperature was –3.817 °C. After the calibration process (Figure 6b,d), the vignette effect improved marginally, with the σ reduced to 0.054 °C and the IQR to 0.030 °C. It is important to note that the bias of the calibrated image was reduced to 0.729 °C and that the pixel temperature values had a narrower distribution than before the calibration.

### 3.3. TeAx 640 Calibration

**Figure 7:**
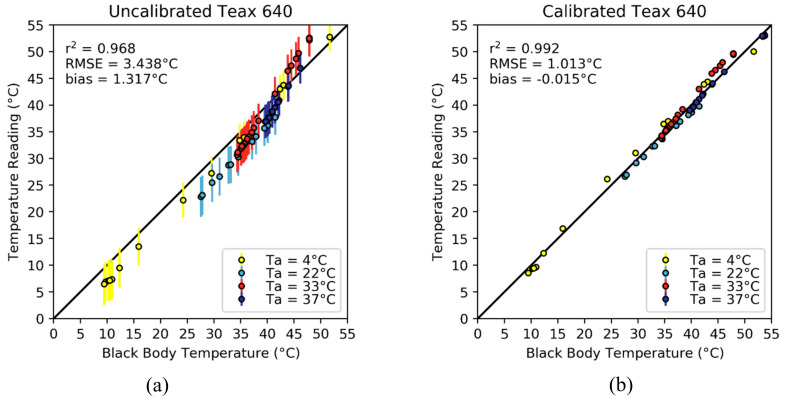
Scatter plot of the average temperature readings of the TeAx 640 camera before (**a**) and after (**b**) calibration. Error bars show the spread of the acquired temperature measurements that add up to the mean bias of the images. The error bars are three times the standard deviation of each sample as a measure of spread. The plots and the statistics were made from the evaluation dataset (n = 70).

The TeAx 640 is an uncooled thermal infrared camera packaged in a way that makes it suitable to mount onto a UAV platform. We applied the methodology of Section 2.4 on a pixel-by-pixel basis using an environmental chamber at four different temperatures during the cooling cycle of the FLIR black body. The uncalibrated thermal measurements had a mean bias across all ambient temperature conditions of 1.317 °C, an RMSE of 3.438 °C and an r^2^ of 0.968. These parameters mean that even though the camera had a high correlation with the actual black body temperatures, it underestimated the actual temperature values of the black body up to approximately 40 °C. At higher black body temperatures, an overestimation was identified (Figure 7a). Furthermore, the high RMSE value would make the camera unsuitable for applications that need a precise temperature value. Additionally, there is significant deviation from the mean value of the images (Figure 7a) shown by the error bars (three times the standard deviation of each image) of each of the validation samples.

After the calibration, the RMSE was reduced by 2.425 °C (from 3.438 to 1.013 °C) across all the environmental chamber temperatures. Once the calibration process was applied, the TeAx 640 had a bias of –0.015 °C, with r^2^ increasing to 0.992. The large difference between the bias (–0.015 C) and RMSE (1.013 °C) implies that the camera calibration process is under- and over-estimating the target temperature in some instances as depicted on Figure 7b. A fitting equation using the mean of all pixel calibration coefficients is shown below:

(11) $$T_{cal}=-0.007\cdot T_{radiometer}^{2}+1.328\cdot T_{radiometer}-0.009\cdot T_{a}+0.288$$

The main contribution to the pixel temperature accuracy came from the uncalibrated measurements ($T_{radiometer}$) in the multilinear regression fit, $\beta_{2}$ being the largest calibration coefficient (1.328). Interestingly, the effect of the ambient temperature on the regression was quite small. On average, a change of 111 °C in ambient temperature was required to affect the calibrated temperature measurement by 1 °C. This is counterintuitive given the frequent assumption of the ambient temperature influence on the vignette effect [34,37,42] that states that the camera response is dependent on the measured object, the camera optics and the FPA temperature (the latter two are directly influenced by the ambient temperature). However, the contribution of the $\beta_{1}$ coefficient to the measured value is small (<0.3 °C on average) for temperature changes below ~33 °C, which are not common during normal field operating conditions. On the other hand, it is reasonable to assume that abrupt changes in the ambient temperature (e.g., by wind speed) have an impact on the FPA stabilization and require additional time for the camera to return to a thermal equilibrium condition. The ambient temperature perturbation response by the thermal cameras was documented in Zhao et al. [40], where they reached a similar conclusion from observed data, i.e., that the camera needs an additional time to reach equilibrium after the FPA and camera optics are cooled/warmed by wind effects.

In the case of the TeAx 640, the vignette effect before the calibration process can clearly be seen in Figure 7a, where the σ and IQR for the uncalibrated images were 1.059 °C and 1.387 °C on average respectively, for the validation samples. After the calibration process, the vignette effect was almost fully compensated, as seen in Figure 7b and by the reduction of the standard deviation and IQR to 0.096 °C and 0.099 °C, respectively.

**Figure 8:**
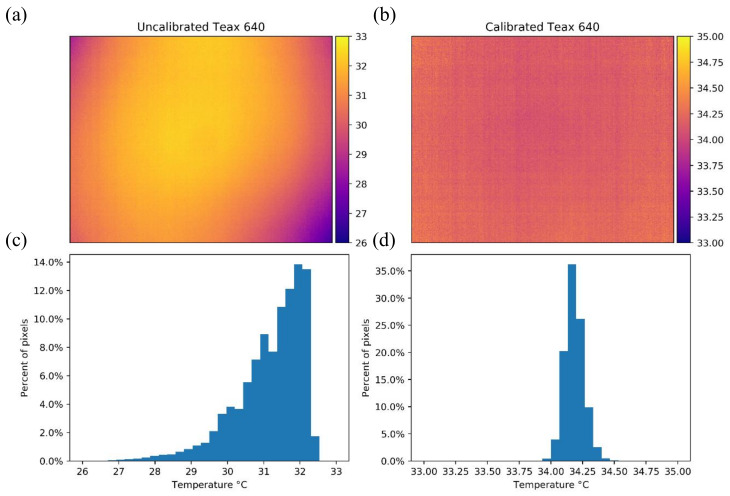
Temperature maps in Celsius and histograms for the TeAx 640 camera while looking at the black body with a temperature of 34.44 °C. Panels (**a**) and (**c**) show the results before the calibration process and panels (**b**) and (**d**) depict the results of the same image after applying the calibration equation for each pixel to the whole image.

An example of the vignette effect is shown in Figure 8a, which is also reflected in the temperature distribution histogram of Figure 8c. Before calibration, the σ and IQR of the image were 1.161 °C and 1.580 °C, respectively. After the calibration process the image had a σ=0.094 °C and IQR = 0.107 °C, which was a significant improvement that translates to a more homogenous image (Figure 8b) and a tighter histogram with more normally distributed temperature measurements (Figure 8d). Additionally, the mean difference from the black body temperature after calibration was 0.772 °C (compared to 4.272 °C before calibration).

### 3.4. Impact of the $\beta_{1}$ and $\beta_{0}$ Calibration Coefficients on the TeAx 640 Vignetting

**Figure 9:**
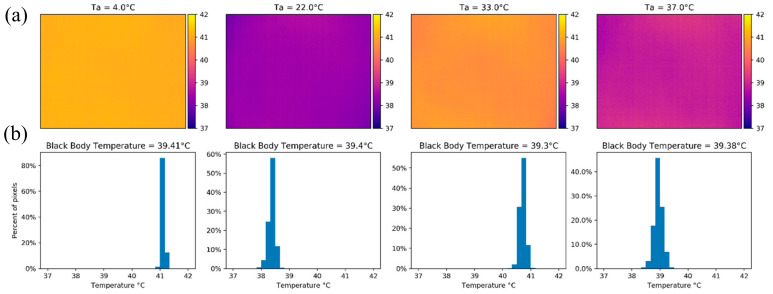
(**a**) Thermal images from the TeAx 640 with the $\beta_{1}$ coefficient set to zero with similar black body temperature under the four ambient temperatures of the calibration experiment. (**b**) The corresponding histograms show an average dispersion lower than 0.106 °C from the mean values (41.13, 38.37, 40.71, 38.95 °C respectively).

To quantify the impact of the $\beta_{1}$ and $\beta_{0}$ calibration coefficients on the vignetting of the TeAx 640 camera, we forced each of them to be zero one at a time (i.e., the coefficient influence was turned off). We chose four images with a similar black body temperature (within <0.1 °C from each other) from each experiment (i.e., one image from when the ambient temperature was 4 °C, 22 °C, 33 °C and 37 °C). When setting the $\beta_{1}$ calibration coefficient to zero (which represents the ambient temperature influence on the calibration process) for the thermal images, most of the corrected image pixel values were concentrated towards the mean, with the vignette effect being most pronounced at 4 and 37 °C based on the temperature distribution of the histograms (Figure 9). In terms of accuracy, given the average low value of the $\beta_{1}$ calibration coefficient (0.009), the average value of the image stayed within the manufacturer’s parameters, having an error lower than 1.5 °C for the tested images.

**Figure 10:**
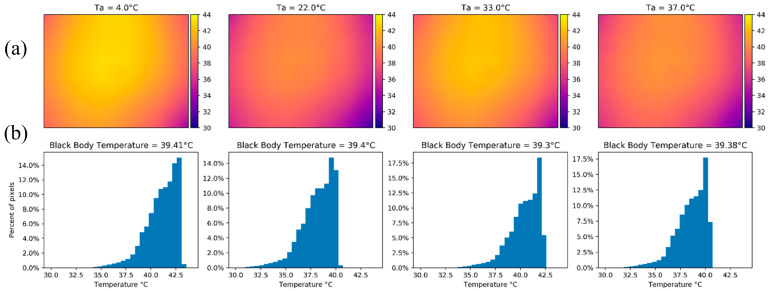
(**a**) Thermal images from the TeAx 640 with the $\beta_{0}$ coefficient set to zero with a similar black body temperature across all ambient temperatures. The vignette effect was present and had the same shape as the uncalibrated images. (**b**) The corresponding histograms are similar to the uncalibrated images with a dispersion of 1.553 °C from the mean values (40.78, 37.88, 40.12, 38.33 °C respectively).

Setting the $\beta_{0}$ coefficient to zero (while leaving the other coefficients active) reintroduced the vignetting effect (Figure 10). While the average value of the measurements did not exceed the 5 °C difference specified by the manufacturer, staying within 1.5 °C of the black body temperature value, the standard deviation of the pixel values was on average 1.553 °C (in contrast to an average of 0.106 °C when the $\beta_{1}$ coefficient was not active).

Table 2 presents the descriptive statistics for when the $\beta_{1}$ and $\beta_{0}$ coefficients were set to zero for each of the evaluated images. Interestingly, the difference between the actual black body temperature and the measured temperature was not larger than 0.4 °C between each scenario. However, when $\beta_{0}$ was set to zero, the standard deviation and IQR of the images were up to 14.8 and 25 times larger, respectively, than when the coefficient was active, which verifies that $\beta_{0}$ has a large influence on mitigating the vignette effect of the images. Furthermore, the measured temperature values had a range of almost 10 °C when $\beta_{0}$ was set to zero as opposed to <1.5 °C when it was active. It also worth noting that the mean and median values were the same when $\beta_{0}$ was active (within 2 significant figures after the decimal point), whereas there was a difference of up to 0.3 °C when set to zero. This small difference between the mean and median values indicates that the vignette effect does not bias the measured temperature if the temperature images are used to get a single average temperature of a homogeneous target.

## 4. Discussion

### 4.1. Instrumentation Requirements of the Calibration Process

Our results showed that the developed radiometric calibration functions for each of the three thermal sensors resulted in significantly improved temperature estimates when evaluated against the black body measurements, along with a reduction of the vignette effect in the case of the thermal cameras. While our approach can be applied to different kinds of thermal infrared sensors, it requires unique temperature radiometric calibration functions to be produced for each sensor. For replication of our calibration approach, we provided a complete list of equipment used for this experiment and associated costs (see Table 1 in Section 2.4 for further details). Most of the equipment used were relatively inexpensive with the exception of the black body and environmental chamber.

While both the Apogee and FLIR A655sc sensors were found to be more sensitive to ambient temperatures, only small impacts were identified for the derived temperature measurements of the TeAx 640 camera. Therefore, an environmental chamber may not be required to produce the radiometric calibration functions for the TeAx 640 camera, which would significantly reduce the costs of equipment required for the calibration process. However, as we let the black body gradually cool down to the ambient temperature, it would be desirable to undertake the calibration process at lower ambient temperatures than the expected operating conditions to expand the range of black body temperature measurements. Without the use of an environmental chamber, it would also be important to ensure a relatively stable ambient temperature and humidity and no wind effects during the data collection process for collection of high-quality calibration data. However, it is important to highlight that the low sensitivity to ambient temperature could vary depending on the thermal camera even within the same models due to size, optics and manufacturing variability. As such, ambient temperature measurements should be as accurate as possible to ensure the reliability of the calibration process.

### 4.2. Ambient Temperature and Vignette Effects

Previous studies have found that the temperature measured by uncooled microbolometer detectors in thermal infrared cameras varies as a function of the temperature of a camera’s FPA, which in turn are affected by ambient temperature [37,61]. While the calibration function for the Apogee radiometer showed that an ambient temperature of approximately 21 °C would cancel out the $\beta_{0}$ offset coefficient and produce a near perfect 1:1 relationship between temperature readings and the black body temperature, a significant bias at low and very high temperatures was identified. On the other hand, the ambient temperature had only a small impact (e.g., 0.30 °C at 33.3 °C) on the UAV-based TeAx 640 measurements. Therefore, based on these observations, ambient temperature may not be as critical a factor for some thermal cameras as previously thought in other studies [62]. While Wolf et al. [63] states that non-uniformity noise (including vignette effect) depends on the FPA temperature, our results (Figure 8) show otherwise, at least after an 80 min warm-up period to ensure stabilization of the camera. Such a long stabilization period may not be practical for all field applications. However, the amount of required warm-up time varies by instrument and it could potentially be shorter. The effects of ambient temperature on a camera’s FPA might to some extent be reduced by the packaging and optics of uncooled thermal infrared cameras, which highlights the importance of suitable insulation for improved camera performance. In fact, Zhao et al. [40] emphasized the importance of proper insulation of the camera lens due to high thermal conductivity from the lens to the FPA. The exposure of the lens to wind during flights may also affect the measurements [24]: work that we are also currently exploring in which we have detected variations in temperature between flight strips. From our laboratory results, which found that different ambient temperatures have a minor impact on our camera accuracy once the FPA has reached thermal equilibrium, we can link these variations to an FPA stabilization process. Insulation of the camera can help to ameliorate the sudden wind effect contribution to the FPA temperature stabilization. However, given that no shielding is perfect a possible solution could be the introduction of known temperature targets to undergo a vicarious calibration process for each flight strip. In addition, it should be noted that the impact of the wind’s cooling on the FPA is also dependent on the camera’s housing and heat dissipation characteristics, which could result in a higher temperature difference between the FPA and the ambient temperature.

Given that the camera response is dependent on camera optics and the FPA temperature, which are directly influenced by the ambient temperature [34,37,42], it would be expected that the ambient temperature would influence the vignette effect. However, our results showed that the vignette effect was primarily affected by the $\beta_{0}$ offset coefficient. Without the $\beta_{0}$ offset coefficient, significant vignette effects were identified (Figure 9). Hence, a major contribution of our thermal radiometric calibration approach is the correction for vignette effects, which are generally assumed to occur because of lens optics and other non-uniformity effects that introduce unrealistic temperature distributions [24]. Similar to Kelly et al. [24] we found that the non-uniformity corrections, performed automatically every 100 frames by the TeAx 640 camera, did not remove vignette effects, emphasizing the need for a vignette correction approach like ours. To further remove vignette effects during the generation of an orthomosaic, it may be advisable to select a blending mode favoring center pixels within each photo. For example, the “Mosaic” blending mode in the Agisoft MetaShape software assigns the highest weight to pixels, where the projection is closest to the normal vector. This means that only the center part of each photo is used in the majority of cases, as long as there is a large overlap between photos. However, as most approaches for producing orthomosaics are based on optical rather than thermal data, further research is needed to produce improved thermal orthomosaics.

### 4.3. Considerations for Field Based Applications

Mesas-Carrascosa et al. [32] emphasized that an accuracy of thermal images greater than 1 ℃ is generally required for applications that require accurate measurements. This highlights the need for careful planning of not just the data collection process, but also of data calibration and correction to reduce errors. Kelly et al. [24] identified a number of sources of error, including radiometric calibration, sensor temperature, vignette effects, non-uniformity noise and atmospheric effects, target emissivity and distance to target. Our ambient temperature dependent radiometric calibration process significantly reduces temperature bias and vignette effects in the acquired imagery under laboratory conditions and provides calibration functions and matrices that are easy to interpret. Given that non-uniformity noise was incorporated into the calibration functions and that sensor temperature may be used as a proxy for ambient temperature after an 80 min stabilization period, only the atmospheric effects, target emissivity and distance to target remain for further correction.

As field-based conditions are more complex than laboratory conditions and introduce a number of additional sources of error in thermal measurements, these require careful consideration as well. Aubrecht et al. [64] identified target emissivity, temperature of surrounding objects reflected by the target object and attenuation of the measured signal by water vapor as important field conditions to consider. Others have found that the atmospheric attenuation of thermal radiation can cause large differences in temperature between the actual and measured temperature [36,65,66]. For example, the temperature signal from vegetation may be contaminated by adjacency effects such as thermal reflections of air and surroundings as well as signal attenuation by water vapor [64]. An example of this could be edge effects along the perimeters of agricultural center pivots, where significant temperature gradients exist, especially in hot climates. The distance between the thermal camera and the target also impact temperature measurements [24].

**Figure 11:**
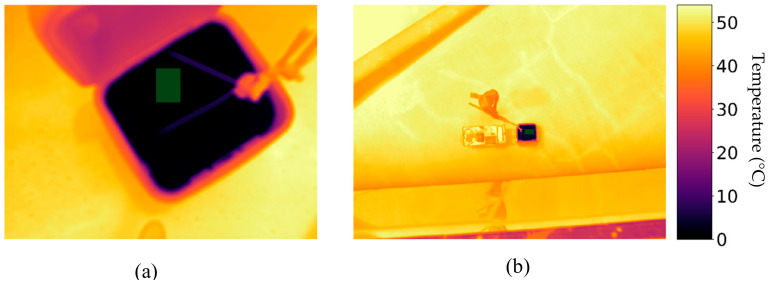
(**a**) Temperature map of a cooler filled with ice taken at a distance of ~40 cm with an ambient temperature of 37 °C. The average value of the ice pixels inside the cooler (represented by the green rectangle) was –0.105 °C. (**b**) Temperature map of same ice filled cooler taken at a distance of ~10m. The average value of ice only pixels (green rectangle) was 4.064 °C illustrating that thermographies can present adjacency effects. The images displayed were collected manually with a FLIR A655sc camera and subjected to the calibration matrices described in this research.

In Figure 11, we provide an example of temperature measurements taken within 5 min of each other at 0.40 and 10 m height from the target. The images were taken around solar noon with a FLIR A655sc camera at an ambient temperature of 37 °C after applying the previously developed calibration matrices. The measured temperature of the ice’s surface had a difference of ~4 ℃. This temperature difference is an example of the adjacency effect, which appears when an adjacent surface, that acts as a radiation source, contributes to the radiation emitted by the observed object [67]. Such a temperature difference could be detrimental if a high accuracy is needed to measure a given object and is an error that can propagate further down the processing chain. As an example, LST needs to undergo a correction for background temperature (taken as the sky temperature) [68], for which it is a common practice to take the temperature of an aluminum plate captured during the UAV survey [69,70,71] (in using such approach, the sky temperature must be within the camera image temperature range). As such, adjacency effects could introduce additional uncertainty for applications that depend on accurate LST values. However, identifying the adjacency effect behavior is complicated and depends on the surface’s properties and structure [72]. Moreover, Zheng et al. [67] identified that the adjacency effects increased with spatial resolution.

In addition to our ambient temperature-dependent radiometric calibration approach for thermal imagery, there are many influences on thermal measurements that still need to be accounted for during field conditions. Most of those are related to atmospheric effects, if an uncooled thermal camera is used from a UAV platform. To correct for local atmospheric effects to estimate land surface temperatures, a vicarious calibration process may be used in combination with our radiometric calibration approach. To undertake vicarious calibration of thermal data, satellite-based studies have used homogenous surfaces such as water bodies and salt flats [73]. For UAV-based campaigns, features that are temperature stable for the duration of a flight mission will suffice, but they should span the full range of temperatures encountered within the area of interest [Kelly et al., 2019]. Hence, a black target to reach high temperatures and a cooler filled with ice (Figure 10) would ensure a large temperature range. Other natural features, such as bare ground might also be used. An Apogee sensor, calibrated with our presented method, would be suitable for collection of ground control temperatures. Also, its proximity to the targets would not be influenced by atmospheric conditions. Based on these observations, an empirical line method [74] could be used to convert UAV imagery to at-surface temperature. Using ground control temperature measurements for all representative surfaces would also benefit in characterizing the possible adjacency effects and the magnitude of their influence [72]. Continuous measurements of temperature control targets with Apogee sensors throughout the duration of a flight mission would document any potential temperature variations, which might be used for estimating error propagation in the derived UAV temperature measurements.

**Figure 12:**
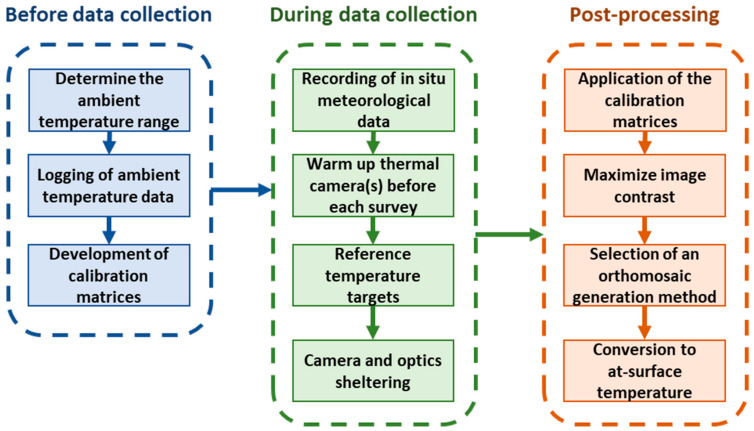
Proposed workflow for conducting a thermal data collection survey. The first step is the derivation of the calibration equations/matrices for the thermal sensor inside an environmental chamber. Next is the surveyed site setup and recording of ancillary data during the thermal data collection. The last step represents the application of the calibration equations/matrices to the collected thermal data, contrast maximization, the selection of an appropriate mosaicking algorithm, and the empirical line method to convert to at-surface temperature based on the reference temperature targets.

A general calibration scheme for UAV and field-derived thermal data would significantly benefit measurements. We have provided a proposed workflow in Figure 12, which involves the derivation of the calibration equations/matrices for each thermal sensor before the field data collection. During the thermal surveys, meteorological data (air temperature, relative humidity and wind speed and direction) should be recorded to apply the calibration and to better evaluate field conditions. The camera should be left to warm up for at least 15 min by powering it on before the survey to avoid adverse stabilization effects on the measurements. Reference temperature targets and/or ground truthing should be placed in the survey area for additional accuracy corrections (such as vicarious calibration). As an additional measure, it is advisable to shield the camera against forced cooling caused by the wind during UAV flight mission to minimize errors due to FPA temperature instability. After the data collection, the previously developed calibration equations/matrices should be applied to the data before further post-processing. We also recommend the application of a linear stretch to the collected imagery based on the minimum and maximum observed temperatures to maximize the contrast in the images [75] before constructing an orthomosaic. Finally, it is important to ensure that any applied orthomosaicing method preserve the physical meaning of the thermal data. There are other effects to consider besides the use of our ambient temperature-dependent radiometric calibration to reduce vignette effects and non-uniformity noise at various temperatures and the conversion to at-surface temperature using temperature control targets. Aubrecht et al. [20] found emissivity to have the greatest influence on measurements of vegetation temperature. However, there are already established procedures in place for determining emissivity of vegetation [76]. Uncooled thermal infrared cameras require an initial stabilization period [24,36], but this issue can be mitigated by warming up the camera well in advance of a flight mission. However, the impact of wind and wind direction on collected thermal imagery requires further research [24,40]. An important first step is to shelter the camera from wind as much as possible [24], although as pointed out by Zhao et al. [40], the sheltering should target the camera optics as well. Wind also changes the microclimate in terms of temperature, heat fluxes and humidity [77], which could introduce an error caused by differences between the ambient temperature around the camera and that of the ground measurements. In situ climate data, such as temperature, wind direction, wind speed and humidity may improve thermal data interpretation and analysis of subsequent outputs (e.g., orthomosaics) from pre-processing routines. Sudden wind gusts during a flight mission may cause significant variations in surface temperature in overlapping images, which will subsequently affect the orthomosaic generation. Additionally, for long surveys the terrain temperature may vary significantly between each UAV transect affecting the agreement of the measured values between the overlapping areas. Further research is required to determine how different orthomosaic approaches impact temperature measurements.

## 5. Conclusions

A novel method for ambient temperature-dependent calibration suited to a variety of uncooled thermal infrared radiometers was developed. The results showed that with a relatively simple laboratory setup, it is possible to establish temperature dependent calibration functions and matrices that can be applied to thermal infrared radiometers (in this case the Apogee SI-11 sensor and FLIR A655sc and TeAx 640 cameras) to significantly reduce vignette effects and increase measurement accuracy. While temperature measurements by the Apogee SI-11 sensor were mainly affected by low and high ambient temperatures, measurement bias and vignette effects in the thermal images collected by the FLIR A655sc and TeAx 640 cameras were significantly reduced when applying the radiometric calibration matrices to correct each pixel. This research clearly showed that there is a need to calibrate thermal imagery, especially to achieve accuracies within 1 ℃. Our research provides a suitable approach for calibrating thermal data immediately after data collection and prior to further image processing in a computationally inexpensive and easy to interpret manner that is dependent on commonly available air temperature measurements. It is recommend that our approach be applied for all thermal sensors and UAV-based cameras prior to or shortly after data collection to develop the calibration matrices before using the data for agricultural applications.

Extensions to our research should focus on determining how often there is a need to undertake ambient temperature-dependent calibration of thermal infrared sensors. Some manufacturers recommend to perform calibration every year. However, the one-year requirement needs to be evaluated by undertaking repeated calibrations on a regular basis. Only then will it be possible to compare the derived calibration functions and matrices of individual cameras to determine when differences become significant over time, and hence should result in sensor recalibration. Our approach forms the initial step in a long line of correction procedures required to accurately measure temperature from thermal imagery. Several of the subsequent correction procedures require further research in order to reach an operational status. For example, the implications of FPA temperature changes due to wind speed and wind direction on UAV-based thermal imagery, both with and without the camera being sheltered during flight missions, needs to be examined. Adjacency effects should also be considered, as they can detrimentally affect the accuracy of the measurements and can propagate further into the application workflow. Finally, there is also a need to assess existing methods for developing a thermal orthomosaic, and potentially identify or develop new blending modes specifically designed for processing thermal data.

## Figures and Tables

**Table 1:** Equipment and associated approximate costs used to develop the temperature radiometric calibration functions for the thermal infrared sensors.

Equipment	Cost (USD)
Instrumentation amplifier	<10
Breadboard (or a printed circuit board)	<10
Resistance Temperature Detector (RTD)	<10
Trim potentiometer	<10
1% resistors (× 6 per RTD)	<10
Laboratory power supply (triple output)	~400
Stirring hotplate (for the RTD calibration process)	~70
Labjack U12 data acquisition card	~200
Black body	~2000–4000 (depending on model)
Wires and solder material	<10
Soldering iron	~70
Environmental chamber	>5000 (depending on size)

**Table 2:** Descriptive statistics to evaluate the impact of the $\beta_{1}$ and $\beta_{0}$ coefficients in the vignetting of the TeAx 640 images. Having the $\beta_{0}$ coefficient active produced images with no apparent vignette effect and with lower standard deviation and interquartile range (IQR) under all evaluated ambient temperatures.

Statistic	$\beta_{1}\mathrm{off}$				$\beta_{0}\mathrm{off}$			
	Ambient Temperature (°C)				Ambient Temperature (°C)			
	4	22	33	37	4	22	33	37
σ(°C)	0.049	0.119	0.104	0.151	1.56	1.60	1.54	1.51
IQR (°C)	0.030	0.074	0.076	0.074	0.77	0.79	0.81	0.72
Mean (°C)	41.11	38.38	40.71	38.94	41.03	38.13	40.36	38.56
Median (°C)	41.11	38.38	40.71	38.94	41.31	38.41	40.37	38.83
Bias (°C)	1.70	–1.02	1.41	–0.44	1.62	–1.27	1.06	–0.82
T_Min_ (°C)	40.76	37.60	39.99	38.10	33.57	30.60	32.81	31.00
T_Max_ (°C)	41.43	38.92	41.19	39.62	43.44	40.62	42.77	41.02
